# QTL-Seq and Transcriptome Analysis Disclose Major QTL and Candidate Genes Controlling Leaf Size in Sesame (*Sesamum indicum* L.)

**DOI:** 10.3389/fpls.2021.580846

**Published:** 2021-02-24

**Authors:** Chen Sheng, Shengnan Song, Rong Zhou, Donghua Li, Yuan Gao, Xianghua Cui, Xuehui Tang, Yanxin Zhang, Jinxing Tu, Xiurong Zhang, Linhai Wang

**Affiliations:** ^1^Oil Crops Research Institute of the Chinese Academy of Agricultural Sciences, Key Laboratory of Biology and Genetic Improvement of Oil Crops, Ministry of Agriculture and Rural Affairs, Wuhan, China; ^2^Zhumadian Academy of Agricultural Sciences, Zhumadian, China; ^3^Xiangyang Academy of Agricultural Sciences, Xiangyang, China; ^4^National Key Laboratory of Crop Genetic Improvement, National Sub-Center of Rapeseed Improvement in Wuhan, Huazhong Agricultural University, Wuhan, China

**Keywords:** sesame, leaf size, QTL-seq, QTL, Transcriptome, candidate genes

## Abstract

Leaf size is a crucial component of sesame (*Sesamum indicum* L.) plant architecture and further influences yield potential. Despite that it is well known that leaf size traits are quantitative traits controlled by large numbers of genes, quantitative trait loci (QTL) and candidate genes for sesame leaf size remain poorly understood. In the present study, we combined the QTL-seq approach and SSR marker mapping to identify the candidate genomic regions harboring QTL controlling leaf size traits in an RIL population derived from a cross between sesame varieties Zhongzhi No. 13 (with big leaves) and ZZM2289 (with small leaves). The QTL mapping revealed 56 QTL with phenotypic variation explained (PVE) from 1.87 to 27.50% for the length and width of leaves at the 1/3 and 1/2 positions of plant height. *qLS15-1*, a major and environmentally stable pleiotropic locus for both leaf length and width explaining 5.81 to 27.50% phenotypic variation, was located on LG15 within a 408-Kb physical genomic region flanked by the markers ZMM6185 and ZMM6206. In this region, a combination of transcriptome analysis with gene annotations revealed three candidate genes *SIN_1004875*, *SIN_1004882*, and *SIN_1004883* associated with leaf growth and development in sesame. These findings provided insight into the genetic characteristics and variability for sesame leaf and set up the foundation for future genomic studies on sesame leaves and will serve as gene resources for improvement of sesame plant architecture.

## Introduction

Sesame is a traditional and ancient oil crop. It has been cultivated for more than five thousand years in Asia and has been considered a high-quality oil crop and health food for humans (Cheng et al., 2006; Namiki, 2007; Takada et al., 2015). Plant leaf represents the main organ for photosynthesis, and the morphology of leaves, such as leaf angle, leaf length, leaf width, leaf size, and leaf shape are crucial factors determining plant architecture, which thereby influence the yield potential (Govaerts et al., 1996; Bar and Ori, 2014). Yield is the consequence of cumulative effects of various traits and processes throughout the whole plant growth period (Mikołajczak et al., 2016). The development of high-yielding sesame varieties with the high-density population is always limited by the plant architecture mainly.

Early studies in other crops suggested that leaf size traits are quantitative traits that ascribe to combinations of multiple genes with a minor effect for each gene (Bian et al., 2014). Moreover, leaf size traits are also strongly influenced by numerous environmental factors (Kobayashi et al., 2003). Recently, QTL related to flag leaf length (FLL), flag leaf width (FLW), and flag leaf area (FLA) have been detected in cereal crops using various populations (Liu et al., 2015). For instance, using six populations of bread wheat across ten different environments, Ma et al. (2019) identified a total of eight major and stable QTL for FLL, FLW, and FLA. Tang et al. (2018) detected 14 and 8 QTL, respectively, for FLL and FLW in rice using a CSSL population. Among these rice QTL, *qFW4-2* was located to a 37-kb region, with the most possible candidate gene which previously detected *NAL1*. Moreover, 17 and 14 QTL, respectively, for FLL and FLW have been identified in a maize-teosinte BC_2_S_3_ RIL population (Fu et al., 2019). Many mutants with leaf size phenotypes have been characterized, and their relevant genes have been cloned in rice (Fujino et al., 2008; Hu et al., 2010; Xiang et al., 2012; Liu et al., 2016). However, few of the identified leaf size QTL have been narrowed down to a small genomic region or cloned (Shen et al., 2012; Takai et al., 2013). In addition, to date, no QTL and candidate genes related to leaf size traits have been reported in sesame.

The genetic mapping in sesame started in 2009 with 220 molecular markers based on RSAMPL (Random Selective Amplification of Microsatellite Polymorphic Loci) (Wei et al., 2009). The recent years witnessed the development and utilization of sesame genomic and genetic resources including a novel genetic map for sesame with 424 SSR genetic markers (Wang et al., 2017a). Genetic markers such as SSR, Indel, AFLP, and RAPD were applied extensively in the QTL mapping of agronomic traits such as yield-related traits, seed, oil quality, and disease resistance (Zhang et al., 2013; Wu et al., 2014; Wang et al., 2016, 2017b). However, it is laborious and time-consuming for genotyping in the mapping population using markers and also hard to directly discover candidate genes and tightly linked markers. The completed genome sequences of cultivated sesame (Wang et al., 2014) enabled the utilization of NGS-based technology in rapid mapping in sesame. The quantitative trait locus (QTL)-seq is a method that combines bulked-segregant analysis (BSA) and high-throughput whole-genome resequencing to detect the major regions of a target quantitative trait in a segregating population (Zhang et al., 2020). Compared with traditional QTL mapping, QTL-seq shows much higher efficiency and accuracy in identifying candidate genomic regions containing QTL affecting target traits in crops. The QTL-seq method has been proved to be successful in many crop populations, such as in the mapping of flowering traits in cucumber (Lu et al., 2014), tomato fruit weight (Illa-Berenguer et al., 2015), rice grain length and weight (Yaobin et al., 2018), purple skin of radish fleshy taproots (Liu et al., 2019), and partial resistance to the fungal rice blast disease and seedling vigor (Takagi et al., 2013).

In this study, we combined QTL-seq, SSR marker mapping, and transcriptome analysis to identify major QTL and candidate genes that shape leaf size in sesame. These results constitute the genetic basis of leaf size traits and will help to improve sesame plant architecture using molecular breeding approaches.

## Materials and Methods

### Plant Materials and Phenotypic Evaluation

A total of 488 of the RIL population were derived from a cross between Zhongzhi No. 13 (female parent with big leaves, numbered BL) and ZZM2289 (male parent with small leaves, numbered SL). The parents and RIL population were grown in three environments (Wuhan, Xiangyang, and Zhumadian, China) in 2017. The three sites are different in climatic conditions, as Wuhan belongs to the Yangtze River valley (Southern China) with a humid subtropical monsoon climate. Zhumadian belongs to the Huang-Huai River valley (northern China) with a warm temperate climate. Xiangyang is in the middle of Wuhan and Zhumadian with a subtropical monsoon climate. The leaf size-related traits, including length of the leaves at the 1/3 position of plant height (TLL), width of the leaves at the 1/3 position of plant height (TLW), length of the leaves at the 1/2 position of plant height (SLL), and width of the leaves at 1/3 position of plant height (SLW), were investigated. Experiments were conducted in a randomized complete block design with three replications, and normal agronomic practices were used in field management. The leaf size-related traits were evaluated in triplicate at the late blossom period in each environment.

### Construction of Extreme Bulks

Flesh leaves of BL, SL, and RIL plants were sampled in the Wuhan field and used for DNA extraction. Each sample DNA was isolated using the CTAB method and used for both QTL-seq and SSR marker analysis. To construct the DNA bulk, 25 top lines were first selected for different traits based on the average phenotypic values from three environments, then each line was checked in different environments to make sure it was included in the extreme groups parallelly. Finally, eight DNA bulks, each had 20 RILs, including long bulk and short bulk for TLL, wide bulk and narrow bulk for TLW, long bulk and short bulk for SLL, and wide bulk and narrow bulk for SLL, were constructed, respectively, by mixing an equal amount of DNAs.

### Generation Analysis of NGS Data

Pair-end sequencing libraries (read length 100 bp) with insert sizes of around 500 bp were prepared for sequencing with the Illumina NovaSeq 6000 PE 150 platform. The filtered high-quality reads were extracted from the raw data and used for further analysis based on a Q-score of 30 across > 90% of bulks. The short reads from DNA bulks were aligned to the Zhongzhi No. 13 reference genome sequence^1^ with BWA software. SNP calling was performed using SAM tools software. Functionally annotated putative SNP detection and candidate polymorphic marker locus annotation were performed with SnpEff software. The SNP-index and Δ(SNP-index) were calculated to identify candidate regions for leaf size QTL. The subtraction of the SNP index of two extreme bulks was used to calculate the Δ(SNP-index). In a given genomic region, an average of SNP-index of SNPs was calculated using a sliding window analysis with 1 Mb window size and 10 kb increment. The Δ(SNP-index) value should not be significantly different from 0 in a genomic region where no major loci were located. Under the null hypothesis of no QTL, the statistical confidence intervals of Δ (SNP-index) for all the SNP positions were conducted with given read depths. Then, they were plotted along with Δ(SNP-index). For each read depth, Δ(SNP-index) in the 95% confidence intervals were obtained following Takagi et al. (2013).

### QTL Analyses With SSR Markers

To confirm the candidate regions for leaf size, we conducted traditional QTL analysis based on SSR markers on the 488 RIL population focusing on the genomic regions detected by the QTL-seq method. The SSR markers in the candidate regions were developed according to Zhongzhi No. 13 reference genome sequence (see text footnote 1). The polymorphic SSR markers in the parents were used to genotype the RILs using the polyacrylamide gel electrophoresis method (PAGE). The genetic map was constructed by the software Join Map 4.0, and genetic distances were estimated with the Kosambi mapping function (Kosambi, 1943). The QTL for leaf size traits were identified by composite interval mapping (CIM) analysis using the Windows QTL Cartographer 2.5 software based on a permutation test (1,000 permutation, *P* = 0.05). Analyses of the QTL interactions across environments were performed using QTLNetwork software version 2.0 with the mixed linear model (MLM) approach (Yang et al., 2008). QTL identified in different environments for the same trait were considered to be the same if their confidence intervals were overlapped. In the present study, a QTL was considered to be major and stable when it was detected in more than two environments and had significant effects accounting for more than 10% of the phenotypic variation.

### RNA Isolation and RNA-seq Analysis

To dissect the gene expression difference between BL and SL, the 8th and 20th new leaves from the bottom of the two parents during plant development were sampled for RNA-seq. Total RNA samples were extracted using Trizol reagent. Three biological replicates of two stages of leaves from the BL and SL were used to create 12 independent RNA libraries. RNA-seq libraries were sequenced on an Illumina Hiseq X ten platform. The gene expression levels were normalized to the number of Reads per Kilobase of transcript per Million mapped reads (RPKM) using the HTSeq 6.0 software (Anders and Huber, 2010).

### Real-Time Quantitative PCR (qRT-PCR) of RNA-seq

Real-time quantitative PCR was performed using SYBR^®^ Select Master Mix (2X) (Vazyme Biotech, Nanjing, China) on a Light Cycler 480 II (Roche, Basel, Switzerland). Specific primers for the candidate genes identified were designed using Beacon Designer 8.0 (Supplementary Table 5). The internal control was the *SIN_1004293, a Histone H3.3* gene which was used to normalize transcript levels. PCR reaction mixtures are as follows: 10 μL mix (Vazyme Biotech, Nanjing, China), 5 μl cDNA, 0.5 μL of each primer, and 4 μL ddH_2_O. The PCR program was conducted according to the protocol of introduction. Pre-incubation 1 cycle: 95°C for 30 s; amplification 40 cycles: 95°C for 10 s, 60°C for 30 s; melting curve 1 cycle: 95°C for 15 s, 60°C for 60 s, 95°C for 15 s; and cooling 1 cycle: 40°C for 30 s. Real-time assay for each gene was performed with three independent biological replicates under identical conditions. The expression levels of gene were calculated from cycle threshold values through the 2-^Δ^ ^Δ^ ^*Ct*^ method (Livak and Schmittgen, 2001).

### Identification of Putative Candidate Genes

The most stable and reliable QTL were mapped on the sesame genome, and the candidate genes located in the confidence intervals were figured out in combination with the gene expression profiles and functional annotation.

## Results

### Phenotypic Variations of Sesame Leaf Size

**FIGURE 1:**
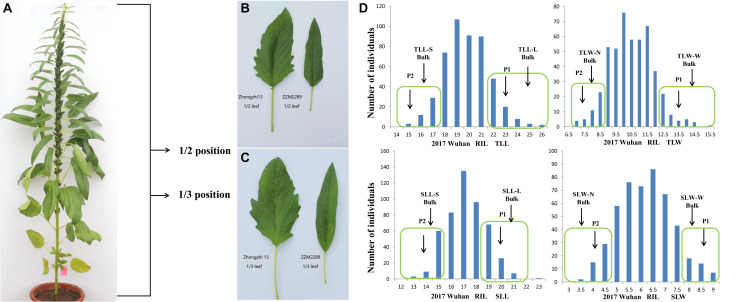
Phenotypic diversity and construction of DNA bulks. **(A)** The whole sesame plant to explain the 1/2 and 1/3 positions of the plant. **(B)** Leaf in the position of 1/2 plant height of two parents. **(C)** Leaf in the position of 1/3 plant height of two parents. **(D)** Frequency distribution of leaf size traits among RIL populations in Wuhan 2017. DNA of 20 RILs with extreme phenotypes was used to develop bulks (L, long S, short N, narrow W, wide).

**FIGURE 2:**
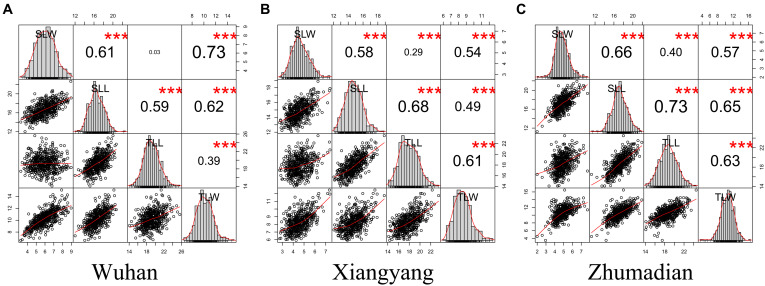
The correlation analysis of leaf size traits. **(A–C)** were the correlation analyses in three environments. ***P < 0.001.

Four leaf size-related traits, including the length and width of the leaves at the 1/3 (TLL and TLW) and 1/2 (SLL and SLW) positions of plant height from the bottom, were investigated in three environments (Figure 1). The leaves of the parent Zhongzhi No. 13 (BL) were more than 32.41% longer and 70.40% wider than those of ZZM2289 (Table 1 and Figure 1). For the RILs, the length and width of the 1/3 position leaf ranged from 14.08 to 25.63 cm and 3.50 to 16.50 cm in the three environments, respectively. For the 1/2 position leaf, the length and width ranged from 11.25 to 22.65 cm and 2.00 to 8.97 cm, respectively. All the target leaf size-related traits showed a continuous variation with a normal distribution characteristic in the three environments (Figure 2). Positive and negative transgressive segregations were generally observed for the four leaf size-related traits.

**TABLE 1:** Leaf size variation of parents and RIL population.

Trait	Environment	Parent		RILs					
		Zhongzhi No. 13	ZZM2289	PD%	Mean	SD	VR	CV%	H^2^
TLL	E1	23.90	15.94	49.93	19.31	1.83	14.08–25.63	9.49	0.92
	E2	20.65	14.95	38.13	17.88	1.67	14.10–23.47	9.36	
	E3	24.50	14.11	73.64	18.70	1.77	14.11–24.50	9.49	
TLW	E1	13.24	7.77	70.40	10.23	1.37	6.60–15.10	13.40	0.89
	E2	10.96	6.30	73.97	8.30	1.18	5.92–12.48	14.17	
	E3	10.88	6.06	79.54	10.53	1.67	3.50–16.50	15.83	
SLL	E1	19.43	14.13	37.51	16.71	1.53	12.02–22.65	9.14	0.85
	E2	17.12	12.93	32.41	14.76	1.14	12.08–18.87	7.70	
	E3	20.20	14.07	43.57	17.20	1.59	11.25–21.90	9.24	
SLW	E1	8.27	3.99	107.27	5.94	1.10	3.24–8.97	18.55	0.87
	E2	6.30	3.23	95.05	4.57	0.78	2.75–7.39	17.08	
	E3	6.61	3.37	96.14	4.86	0.76	2.00–7.71	15.65	

*PD, percentage difference between the two parents. VR, variation range. SD, standard deviation. CV, coefficient of variation. H^2^, heritability. E1, Wuhan E2, Xiangyang E3, Zhumadian.*

The four leaf-related traits had significant correlation coefficients with each other in different environments, except for SLW and TLL in Wuhan (Figure 2). All four traits had broad-sense heritability (H^2^) over 0.85 (Table 1). The ANOVA showed that environment effects were significant for TLL, TLW, SLL, and SLW at *P* < 0.001.

### QTL-seq Identified 77 Candidate Genomic Regions Controlling Sesame Leaf Size

Based on the mean values of leaf length and width in the three environments, four pairs of extreme separated DNA bulks were constructed using the leaves at the 1/3 (TLL and TLW) and 1/2 (SLL and SLW) positions of plant height (Figure 1D). 10 samples in total (two parents and eight extreme bulks) were then analyzed using QTL-seq based on Illumina NovaSeq 6000 PE 150 platform. For each sample, 96,122,878, 46,240,582, 121,519,676, 124,032,266, 127,961,366, 120,295,522, 123,472,918, 118,170,232, 121,852,830, and 120,478,184 clean short reads from different samples were generated (100 bp in length), respectively. Among these short reads, 69.05–81.93% were uniquely and confidently mapped to the reference genome version 1.0 of Zhongzhi No. 13. 580,091, 582,745, 605,167, 585,644, 587,976, 587,264, 584,329, and 584,571 genome-wide SNPs were identified, respectively, for each bulk (Supplementary Table 1).

**FIGURE 3:**
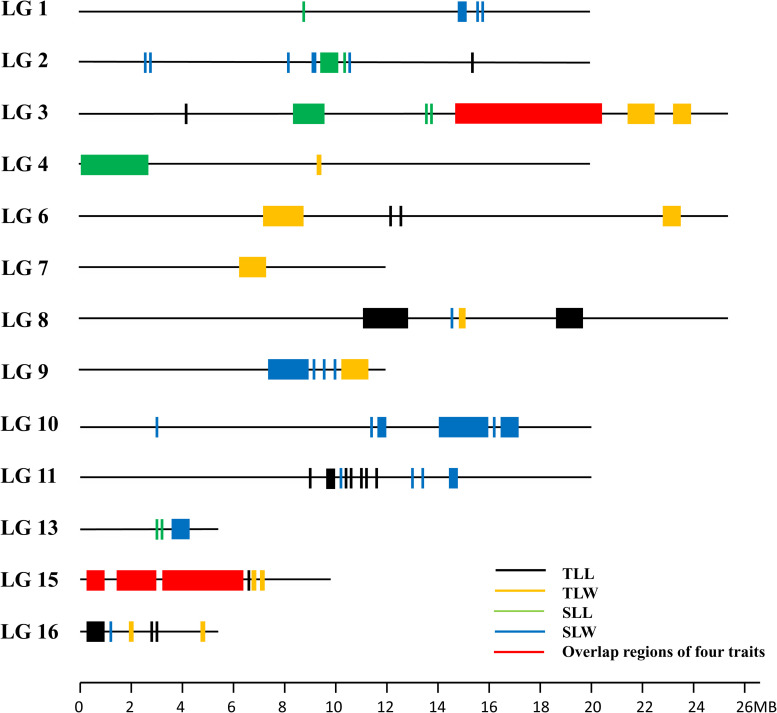
The distribution of genomic regions detected by QTL-seq.

**FIGURE 4:**
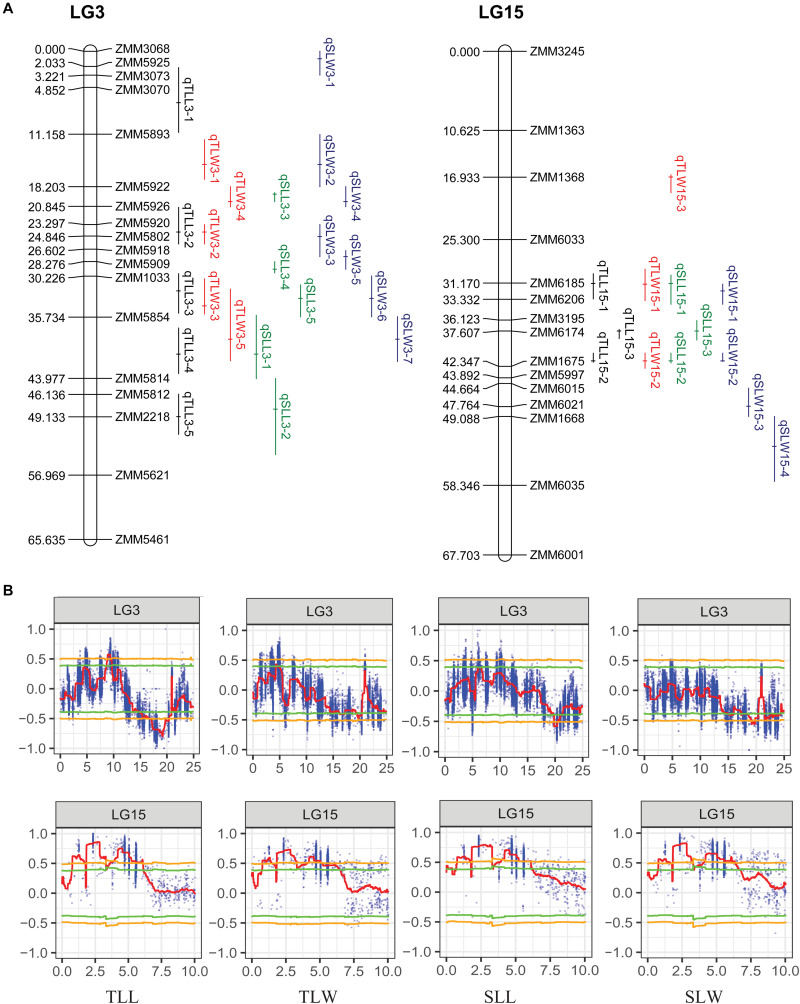
The genomic regions detected by QTL-seq and linkage maps of LG3 and LG15. **(A)** The genetic linkage map of LG3 and LG15. **(B)** The genomic regions detected by QTL-seq on LG3 and LG15.

The ΔSNP-index was calculated by combining the information of the SNP index for each pair of extreme bulks. At the 95% significance level, a total of 77 candidate genomic regions that may contain the QTL of leaf size were figured out on the 13 linkage groups (LGs) except for LG5, LG12, and LG14. LG2, LG3, LG10, LG11, LG15, and LG16 contained 6 or more candidate regions (Figure 3). It was worth noting that there were some pleiotropic loci on LG3 and LG15 (Figure 4). Moreover, we detected 823,202 SNPs within the intervals of the 77 candidate genomic regions (Supplementary Table 2). These SNPs were further targeted for sesame leaf size candidate gene identification.

### QTL Mapping for Sesame Leaf Size Using SSR Markers

To specifically detect QTL linked to sesame leaf size-related traits, 942 SSR markers (including 62 published and 880 newly developed) in the intervals of the 77 candidate genomic regions revealed by QTL-seq were tested in the two parents. Among the developed SSR markers, 81 were polymorphic between the parents, BL and SL, accounting for 8.6% of the total markers. The 81 polymorphic SSR markers were used to genotype the 488 RILs, and a genetic linkage map composed of 71 SSR markers was constructed using Join map 4.0 for further QTL mapping (Supplementary Table 3). The composite interval mapping (CIM) method detected 56 QTL underlying the four leaf-related traits across three environments (Table 2). Meanwhile, 14 QTL explained 1.87–13.43% of phenotypic variation (PVE) for TLL; 11 QTL explained 1.87–27.50% PVE for TLW; 13 QTL explained 2.08–21.13% of PVE for SLL; and 18 QTL explained each 1.91–16.95% of PVE for SLW. The significant LOD score ranged from 2.38 to a high of 30.33. 15 of the 56 QTL were detected in multiple environments, among which, there were seven major QTL with more than 10% PVE, *qTLL15-1*, *qTLW15-2*, *qTLW15-1*, *qSLL15-1*, *qSLL15-2*, *qSLW15-1*, and *qSLW15-2*. These seven main effect QTL were all mapped on LG15. Alleles from Zhongzhi No.13, a big leaf parent, at all of mapped major QTL had increasing effects for leaf size.

**TABLE 2:** Detail information about QTL in RIL population.

QTL	LG	Maker interval	Environment	Peak position (cM)	Range (cM)	LOD score	PVE%	Addictive effect
qTLL2	2	ZMM6151–ZMM3937	E1	0.01	0–3.6	4.33	2.89%	−0.43
			E2	0.01	0–4.8	3.46	2.24%	−0.32
qTLL3-1	3	ZMM3070–ZMM5893	E1	6.91	2.9–10.9	2.38	2.08%	−0.29
			E3	4.91	3.0–7.5	4.19	3.07%	−0.38
qTLL3-2	3	ZMM5920–ZMM5918	E1	24.31	21.8–25.9	3.61	2.55%	−0.33
			E2	25.91	23.7–27.6	3.43	2.47%	−0.30
qTLL3-3	3	ZMM1033–ZMM5854	E2	32.21	30.1–35.2	6.53	5.00%	−0.38
qTLL3-4	3	ZMM5854–ZMM5814	E2	40.71	38.4–43.3	8.80	7.79%	−0.47
			E3	39.71	35.7–43.3	6.62	5.89%	−0.54
qTLL3-5	3	ZMM5812–ZMM5621	E3	49.11	48.0–51.1	6.36	4.73%	−0.47
qTLL6	6	ZMM2202–ZMM4687	E1	16.41	6.1–27.3	2.55	1.87%	0.28
qTLL8	8	ZMM1682–ZMM5657	E1	0.01	0–18.0	2.96	1.98%	0.29
qTLL11-1	11	ZMM1812–ZMM6086	E1	25.41	20.3–31.8	7.12	8.17%	0.58
qTLL11-2	11	ZMM1809–ZMM1812	E2	19.31	14.3–25.7	5.32	3.47%	0.32
qTLL13	13	ZMM6126–ZMM6124	E1	23.61	23.0–23.7	9.12	6.86%	0.75
qTLL15-1	15	ZMM6185–ZMM6206	E1	31.21	30.2–32.2	18.12	13.43%	0.74
			E2	32.21	30.7–33.3	12.72	9.09%	0.53
			E3	31.21	29.9–32.6	7.80	5.81%	0.54
qTLL15-2	15	ZMM6174–ZMM5997	E1	41.61	40.6–41.8	8.70	9.25%	0.62
qTLL15-3	15	ZMM6174–ZMM1675	E2	37.61	37.4–38.6	6.78	4.67%	−0.39
			E3	37.61	37.6–39.0	3.07	2.42%	−0.36
qTLW3-1	3	ZMM5893–ZMM5922	E1	15.21	13.0–17.2	7.60	6.78%	−0.38
qTLW3-2	3	ZMM5920–ZMM5802	E1	24.31	23.6−25.9	9.26	6.23%	−0.36
			E2	25.91	24.9−27.6	8.27	6.47%	–0.31
		E3	24.91	24.3−26.6	4.30	3.42%	–0.33	
qTLW3-3	3	ZMM1033–ZMM5854	E1	34.21	31.8−35.3	6.02	4.47%	−0.31
qTLW3-4	3	ZMM5922–ZMM5926	E2	20.21	18.40–20.90	6.55	5.15%	−0.28
qTLW3-5	3	ZMM5854–ZMM5814	E3	38.71	33.60–41.60	7.72	7.41%	−0.49
qTLW6	6	ZMM4687–ZMM4686	E1	21.31	20.30–23.70	5.62	3.36%	−0.27
qTLW13	13	ZMM6127–ZMM6124	E2	23.61	23.00–23.70	2.68	1.87%	0.20
qTLW15-1	15	ZMM6185–ZMM6206	E1	31.31	29.3–32.4	30.33	27.50%	0.76
			E2	32.21	30.5–33.4	26.79	21.36%	0.55
			E3	32.21	31.4–33.5	16.44	14.20%	0.67
qTLW15-2	15	ZMM6174–ZMM1675	E1	41.61	40.6–41.7	7.60	8.21%	0.42
			E2	41.61	40.6–41.9	8.94	10.06%	0.37
			E3	42.41	41.5–42.6	7.94	7.05%	0.47
qTLW15-3	15	ZMM1363–ZMM1368	E2	16.91	16.6–19.0	2.55	2.21%	0.18
qTLW16	16	ZMM2897–ZMM6141	E1	0.01	0–1.5	8.46	5.43%	−0.34
qSLL2	2	ZMM3937–ZMM6160	E1	12.61	3.2–21.8	2.66	2.99%	−0.27
qSLL3-1	3	ZMM5854–ZMM5814	E1	40.71	35.8–44.0	3.26	2.87%	−0.26
			E2	38.71	37.2–41.6	8.71	8.70%	−0.34
			E3	38.71	36.7–41.5	9.05	8.89%	−0.47
qSLL3-2	3	ZMM5814–ZMM2218	E1	48.11	45.2–54.2	4.40	3.08%	−0.27
			E3	45.01	44.0−46.0	6.61	5.45%	−0.37
qSLL3-3	3	ZMM5922–ZMM5926	E2	19.21	19.2−20.0	7.13	6.45%	–0.29
qSLL3-4	3	ZMM5909–ZMM1033	E2	29.31	28.3−29.8	9.29	8.22%	−0.33
qSLL3-5	3	ZMM1033–ZMM5854	E3	33.21	31.6−35.7	8.68	8.17%	−0.46
qSLL6-1	6	ZMM4689–ZMM2202	E1	9.01	1.9−13.4	4.80	4.37%	−0.32
qSLL6-2	6	ZMM2202–ZMM4686	E1	21.31	20.4–25.2	6.28	4.14%	−0.32
qSLL11	11	ZMM1813–ZMM1809	E3	18.41	14.5−27.1	3.12	2.49%	0.25
qSLL15-1	15	ZMM6185–ZMM6206	E1	31.21	30.3−32.3	28.83	21.13%	0.71
			E2	33.31	33.3−34.0	14.27	11.93%	0.40
			E3	31.21	30.3−33.4	8.78	6.82%	0.43
qSLL15-2	15	ZMM6174–ZMM1675	E1	41.61	40.6−41.8	10.22	11.25%	0.52
			E2	41.61	40.6−41.9	7.77	8.74%	0.34
qSLL15-3	15	ZMM3195–ZMM6174	E3	37.61	36.2−38.8	3.70	3.02%	−0.29
qSLL16	16	ZMM2897–ZMM6141	E1	0.01	0–1.7	3.21	2.08%	−0.22
qSLW2	2	ZMM6151–ZMM3937	E2	0.01	0–4.3	8.84	6.21%	0.23
qSLW3-1	3	ZMM3068–ZMM5925	E1	1.01	0–3.2	3.31	3.37%	−0.20
qSLW3-2	3	ZMM5893–ZMM5922	E1	15.21	12.8–18.2	4.04	3.92%	−0.22
qSLW3-3	3	ZMM5802–ZMM5918	E1	24.91	23.4–27.6	3.00	1.91%	−0.15
qSLW3-4	3	ZMM5922–ZMM5926	E2	20.21	18.7–20.9	7.34	5.85%	−0.19
			E3	19.21	18.2–20.6	8.32	7.72%	−0.21
qSLW3-5	3	ZMM5918–ZMM5909	E2	27.61	27.2–29.3	9.83	7.75%	−0.22
			E3	29.31	28.9–29.9	10.64	9.61%	−0.23
qSLW3-6	3	ZMM1033–ZMM5854	E2	33.21	32.6–35.7	7.61	6.69%	−0.20
qSLW3-7	3	ZMM5854–ZMM5814	E3	38.71	35.7–41.7	6.56	6.46%	−0.19
qSLW6	6	ZMM2202–ZMM4686	E1	21.31	20.4–23.8	8.96	6.09%	−0.28
			E3	25.31	21.3–27.3	3.35	3.00%	0.13
qSLW10	10	ZMM1451–ZMM3461	E2	19.91	16.9–20.9	3.82	2.98%	−0.13
			E3	19.91	9.8–20.9	2.85	2.47%	−0.12
qSLW11-1	11	ZMM4689–ZMM1813	E1	12.71	9.1–14.9	5.95	3.95%	−0.22
qSLW11-2	11	ZMM1812–ZMM6086	E1	28.41	19.3–39.7	3.02	3.69%	−0.21
qSLW13	13	ZMM6122–ZMM6127	E1	0.01	0–6.2	4.24	3.05%	0.19
			E2	0.01	0–5.5	3.16	2.30%	0.12
qSLW15-1	15	ZMM6185–ZMM6206	E1	32.21	31.3–34.0	22.31	16.95%	0.48
			E2	30.31	29.1–32.9	5.81	6.20%	0.22
			E3	32.21	31.6–33.3	13.40	11.44%	0.26
qSLW15-2	15	ZMM6174–ZMM1675	E1	41.61	40.6–41.7	9.63	10.56%	0.41
			E2	41.61	40.6–41.9	7.83	8.26%	0.22
			E3	42.41	41.0–52.9	5.42	4.52%	0.16
qSLW15-3	15	ZMM6015–ZMM6021	E2	47.71	45.3–49.1	3.82	2.68%	0.15
qSLW15-4	15	ZMM1668–ZMM6035	E2	53.11	49.1–57.8	3.87	3.77%	0.17
qSLW16	16	ZMM2897–ZMM6141	E1	0.01	0–1.6	4.80	3.17%	−0.20

*E1, E2, and E3 represented Wuhan, Xiangyang, and Zhumadian, respectively. Range, the confidence interval of QTL position. PVE, the percentage of phenotypic variance explained by a single QTL.*

Although 56 QTL for the leaf related traits were mapped on nine linkage groups, they were not evenly distributed. Among the 56 QTL detected in the RIL population, 35 were clustered on LG3 and LG15, and some pleiotropic loci were also concentrated on these two linkage groups, confirming the result of QTL-seq (Figure 4). The QTL *qTLL15-1*, *qTLW15-1*, *qSLL15-1*, and *qSLW15-1* that were clustered in the same region between markers ZMM6185 and ZMM6206 were all detected across three environments with the highest phenotype contribution rates for different traits ranging from 5.81 to 27.50% averaged 13.82%. The pleiotropic region of different leaf size parameters was designated *qLS15-1*.

### Epistasis Analysis

Twelve pairs of loci with significant (*P* < 0.05) additive × additive epistatic effects regulating four sesame leaf size-related traits were detected (Table 3). The phenotypic variance explained by each pair of epistatic interactions ranged from 0.14% to 0.80%, and the epistatic interactions accounted for 5.26% of the phenotype variation totally. Three interactions occurred between two loci on the same linkage group, while others on separate linkage group. Six of 12 pairs involved major QTL locus with a negative value indicated that the parental two-locus genotypes have a negative effect and that the recombinants had a positive effect. Furthermore, significant epistatic QTL × environment (AAijE) effects for three of the 12 epistatic QTL pairs were identified, and these AAE effects differed considerably in direction under different environments.

**TABLE 3:** Epistatic effect of QTL for leaf size-related traits identified in RIL population.

Trait	^*a*^LG-Ini	Flanking markers	^*B*^mQTLi	^*a*^LG-Inj	Flanking markers	^*b*^mQTLj	^*c*^AAij	^*c*^AAijE1	^*c*^AAijE2	^*c*^AAijE3	^*d*^H^2^(AAij)%	^*d*^H^2^(AAijE)%
TLL	2–1	ZMM6151–ZMM3937		15–4	ZMM6035–ZMM6001		−0.20***				0.45	
	3-9	ZMM5802–ZMM5918		15-5	ZMM6185–ZMM6206	qTLL15-1	−0.10*				0.23	
TLW	3-9	ZMM5802–ZMM5918		15-5	ZMM6185–ZMM6206	qTLW15-1	−0.16***	0.23**		−0.28***	0.59	0.66
	15-5	ZMM6185–ZMM6206	qTLW15-1	16-1	ZMM2897–ZMM6141		−0.12**				0.33	
	15-5	ZMM6185–ZMM6206	qSLL15-1	15–14	ZMM6035–ZMM6001		−0.28***				0.8	
SLL	15-16	ZMM6206–ZMM3195		15-8	ZMM6174–ZMM1675	qSLL15-2	−0.41***				0.75	
	2-1	ZMM6151–ZMM3937		6-2	ZMM2202–ZMM4686		−0.06**		0.29***	−0.28***	0.14	0.60
SLW	2-1	ZMM6151–ZMM3937		16-1	ZMM2897–ZMM6141		0.05*				0.14	
	3-10	ZMM5918–ZMM5909		3-13	ZMM5854–ZMM5814		0.15***	0.08*			0.67	0.71
	3-10	ZMM5918–ZMM5909		10-2	ZMM1451–ZMM3461		−0.07**				0.27	
	3-10	ZMM5918–ZMM5909		15-5	ZMM6185–ZMM6206	qSLW15-1	−0.07**				0.27	
	4-1	ZMM4980–ZMM4977		15-2	ZMM1363–ZMM1368		0.09***				0.62	

*^a^LG-Ini and LG-Inj represented the linkage group number-interval of the points being tested in the analysis. ^b^mQTLi and mQTLj were major QTL involved in interaction. ^c^AAij and AAijE were additive × additive effect interaction and the interaction effect of epistatic QTL by environment, respectively. ^d^H^2^(AAij) and H^2^(AAijE) represented the phenotypic variance explained by AA and AAE, respectively. ^∗^Significant at P < 0.05, ^∗∗^significant at P < 0.01, ^∗∗∗^significant at P < 0.001.*

### The Candidate Genes for the Major Stable Locus qLS15-1

The major stable locus *qLS15-1* was mapped in the sesame genome between 2,339,824 and 2,747,783 bp on LG15. The genomic region spanning 408 Kb had a total of 55 annotated or predicted gene models. We examined the 46,464 SNPs and detected that only six missense variant SNPs were located in the *qLS15-1* locus. Two genes, *SIN_1004921* and *SIN_1004909*, were found to be under the influence of these six SNPs.

RNA-seq provided the gene expression profiles between two parents during leaf development. Among the 55 genes, 12 expressed lower and 14 expressed higher in the large leaf parent at both the 8th and 20th leaf stages. The gene expressions were validated using qRT-PCR by selecting eight genes. It showed that the expression profiles of the eight genes from qRT-PCR were consistent with RNA-seq with similar trends, which thus supported the validity of RNA-seq analysis (Supplementary Figure 1). The expression profiles of the two genes influenced by missense variant SNPs showed no difference between BL and SL. Therefore, they cannot be included in the list of candidate genes for sesame leaf size.

**FIGURE 5:**
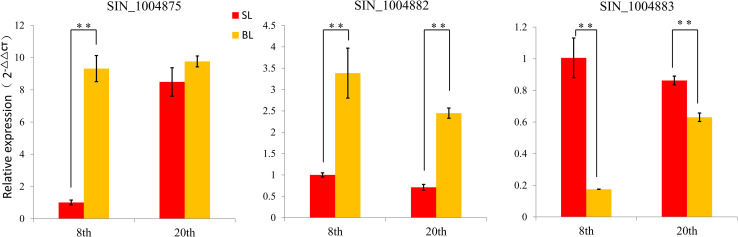
Comparison the relative expression of three candidate genes between BL and SL at different stages by qRT-PCR. Error bars indicate standard deviations (SD) based on two replicates. **P < 0.01, *t* test.

In combination with the predicted gene function and expression profiles, we selected three genes (*SIN_1004875*, *SIN_1004882*, and *SIN_1004883*) that control sesame plant leaf size for further study (Supplementary Table 5). Both RNA-seq and qRT-PCR showed the differences of the three genes in BL and SL (Figure 5). Function annotation showed that the three genes were known to regulate cell proliferation and expansion by affecting cell wall biosynthesis, auxin transportation, and cell cycle. These results suggested the three candidate genes involved in the regulation of sesame leaf size.

## Discussion

Leaf size is an important component of plant architecture and further influences yield potential (Amalero et al., 2003; Tambussi et al., 2007; Jiang et al., 2013). In rice (*Oryza sativa L*), the top three leaves were identified as the most essential photosynthesis organs and the main source of carbohydrate accumulation (Li et al., 1998; Khaliq et al., 2008; Liu et al., 2015; Cui et al., 2017). Sesame yield is lower than 1,500 kg/ha usually, and the ideal plant architecture is sure to be the key factor for yield improvement. Like maize, rice, and wheat which went through the “green revolution,” breeding sesame varieties with ideal plant architecture (small leaf size, small leaf angle, and semi-dwarf plant) might improve sesame yield components and resistance to diverse stresses (Baydar, 2005; Wang et al., 2016). In the early study, leaf size traits had been demonstrated to be quantitative traits (Simon, 1999; Himanen et al., 2007). Sesame leaf traits were more complicated and few studies on it had been reported, especially for the large population, in consideration of the difference of the leaf at different positions from bottom to top. In the present study, the leaves at the middle and one-third positions of plant from the bottom were investigated based on sesame specific plant architecture with small leaves at top position but large leaves at middle and lower positions, which were the key determinants of sesame plant architecture and sunlight capture ability. The results showed though that sesame leaf size was easily affected by the environmental conditions and had broad variation but still had high heritability (all the H^2^ were higher than 0.85; Table 1). It suggested that the detection, validation, and clone of QTL for leaf size-related traits should be based on multiple environments to increase reliability.

QTL-seq combined with conventional marker mapping has been proved to increase the efficiency and accuracy of identification of genomic regions harboring the major QTL in several crops including Chinese cabbage (Li et al., 2020), peanut (Luo et al., 2019), cucumber (Lu et al., 2014), and chickpea (Deokar et al., 2019). To our knowledge, this study represents the first report on the genetic control of leaf size in sesame. QTL-seq firstly predicted 77 candidate genomic regions related to sesame leaf width and length. Targeting these genomic regions, 942 SSR markers were used to scan the two parents, among which 81 (8.6%) of them were polymorphic. The similar low polymorphism of SSR markers in sesame had been reported previously. Zhang found that 6.52% of SSR markers were polymorphic in a mapping population (Zhang et al., 2012). Wang designed and synthesized 1550 SSR markers from transcript sequences, and only 59 (3.8%) showed polymorphism within 36 individuals (Wang L. et al., 2012). Wang also found 6.8% of 7357 SSR markers amplified different bands in two materials (Wang et al., 2017b). The low polymorphism of SSR markers in sesame is likely due to its narrow genetic basis (Dixit et al., 2005; Wei et al., 2008).

We used the 81 polymorphic SSR markers to map the sesame reference genome and identified 56 QTL for leaf size in sesame. The PVE of 56 mapped QTL varied from 1.87 to 27.50%. Some QTL clustered on LG2, LG3, LG6, LG11, LG13, LG15, and LG16, suggesting several loci with a pleiotropic effect for different sesame leaf size traits. Particularly, two noticeable loci on LG15 contained four and six QTL, respectively, with environmentally stable expression and higher PVE. The one in the interval between SSR markers ZMM6185 and ZMM6206, namely, *qLS15-1* contained four QTL, and each of them was detected in three environments with the maximum 27.50% of PVE. The other in the interval between ZMM6174 and ZMM1675 contained six QTL, and two of them can be detected in multiple environments with the maximum of 11.25% of PVE. Co-localization may suggest pleiotropy or close linkages whereby a genomic region contains a cluster of QTL that affect some traits indicating the benefit for improvement breeding efficiency for multiple elite traits (Wang P. et al., 2012). Many studies had reported the co-located QTL for leaf size traits (Wu et al., 2016; Hussain et al., 2017; Du et al., 2019). A QTL which consistently expresses in different genetic backgrounds and environments with a large effect is theoretically regarded as a valuable locus for further analysis such as fine mapping and cloning (Bonneau et al., 2013). Further fine mapping and cloning of these QTL to determine their genetic functions and physical relationships will improve our understanding of leaf size development. Clustered QTL regions will be targeted to understand molecular mechanisms involved in sesame leaf development and to improve sesame plant architecture.

Epistatic interactions have been proved to influence the many phenotype of morphology and yield related traits (Wu, 2012; Hou et al., 2015). Twelve pairs of loci with significant additive × additive epistatic effects regulating four sesame leaf size-related traits were detected. Although the effects of epistasis varied considerably among the traits, the total effects of the epistatic QTL (5.26%) were much smaller than those of the major QTL, indicating that additive effects contributing to variation in sesame leaf size are predominated in our population. These results demonstrated that epistasis could be of minor importance to variation in sesame leaf size development.

Various factors, such as plant hormones and transcription factors, can elaborate the morphology and size of the leaf by regulating cell division and cell expansion (Andriankaja et al., 2012; Gonzalez et al., 2012; Powell and Lenhard, 2012). In consideration of the pleiotropic and stable high PVE of *qLS15-1*, we selected this locus for candidate gene identification by comparing BL and SL transcriptomes at two stages of sesame development. Among the 55 genes in *qLS15-1*, 26 expressed differently and 29 showed similar expression profiles in the two parents at both the 8th and 20th leaf stages. The 29 similar expressed genes, including the two genes identified by examining the SNPs which may suggest other type mutations such as insert, deletion, structure variation, or mechanisms such as alternative splicing and so on, will be involved in the regulation of sesame leaf size development. Based on their expression profiles and functional annotation, three candidate genes *SIN_1004875*, *SIN_1004882*, and *SIN_1004883* were identified associated with leaf development in sesame. *SIN_1004875* is homologous to *IRX14-L* (*AT5G67230*), which encodes a member of the glycosyltransferase 43 family (*GT43*) involved in the synthesis of the hemicellulose glucuronoxylan, a major component of secondary cell walls (Lee et al., 2010; Wu et al., 2010). This gene may function for cell expansion by synthesizing new cell wall material to counteract the loosening of the cell wall and fuel further leaf growth (Powell and Lenhard, 2012). *SIN_1004882* is homologous to *Cyclin-D3-3* (*AT3G50070*), which is a member of D3-type cyclin (*CYCD3-1*, *CYCD3-2*, and *CYCD3-3*). Studies showed that *CYCD3* can affect cell number and cell size in developing leaves by regulating the duration of the mitotic phase and timing of the transition to endocycles (Dewitte et al., 2007). *SIN_1004883* is homologous of *ADP-ribosylation factor 1* (*AT1G23490*), a gene that was found to correlate with auxin directional transportation by recycling *PIN* auxin transporters and participating in various auxin-dependent developmental processes in *Arabidopsis* (Tanaka et al., 2014). Collectively, the three candidate genes were worthy to be further validated by transgenics or gene editing. The study also suggested the reliability of QTL-seq combined with conventional marker mapping in sesame not only is labor-saving and time-saving for sesame QTL mapping but also can detect minor loci.

## Conclusion

This study detected 56 QTL controlling sesame leaf length and width with PVE from 1.87 to 27.50% across three environments. The stable pleiotropic locus *qLS15-1* presented with the maximum 27.50% phenotype contribution rates for different leaf width and length. Three candidate genes (*SIN_1004875*, *SIN_1004882*, and *SIN_1004883*) that may control sesame leaf development were figured out in combination with the gene function annotation and expression profiles. The study provided the first utility of QTL-seq combined with SSR marker mapping and comparative transcriptome analysis on sesame quantitative traits and revealed the genetic variations and candidate loci and genes underlying sesame leaf sizes. These findings may serve as genetic resources for improvement of sesame plant architecture and yield.

## Data Availability Statement

The datasets presented in this study can be found in online repositories. The names of the repository/repositories and accession number(s) can be found below: GeneBank and SRR12341778∼SRR12341789.

## Author Contributions

LW, XZ, and JT contributed to the design of the research. CS, XC, XT, YZ, and LW investigated the phenotypes. SS, CS, RZ, YG, and DL prepared the leaf samples. SS and CS participated in locus and gene annotation. LW and CS performed statistical analysis and wrote the manuscript. All authors read and approved the final manuscript.

## Conflict of Interest

The authors declare that the research was conducted in the absence of any commercial or financial relationships that could be construed as a potential conflict of interest.

## Footnotes

1 http://www.ocri-genomics.org/Sinbase/

## Supplementary Material

Supplementary Figure 1 Comparison of the relative expression abundance measured by qRT-PCR and RNA-seq for 8 selected genes between BL and SL at different stages.

Supplementary Table 1 Statistical table of sequencing in different bulks.

Supplementary Table 2 Number of SNPs in candidate regions detected by QTL-seq.

Supplementary Table 3 The information of the 71 SSR markers.

Supplementary Table 4 Candidate genes in the *qLS15-1*.

Supplementary Table 5 The primer sequence for qRT-PCR.
