# Supplementary figures and images for: QTL-Seq and Transcriptome Analysis Disclose Major QTL and Candidate Genes Controlling Leaf Size in Sesame (Sesamum indicum L.)
Source: Front Plant Sci. 2021 Feb 24;12:580846. doi: 10.3389/fpls.2021.580846 (PMC7943740; doi:10.3389/fpls.2021.580846)

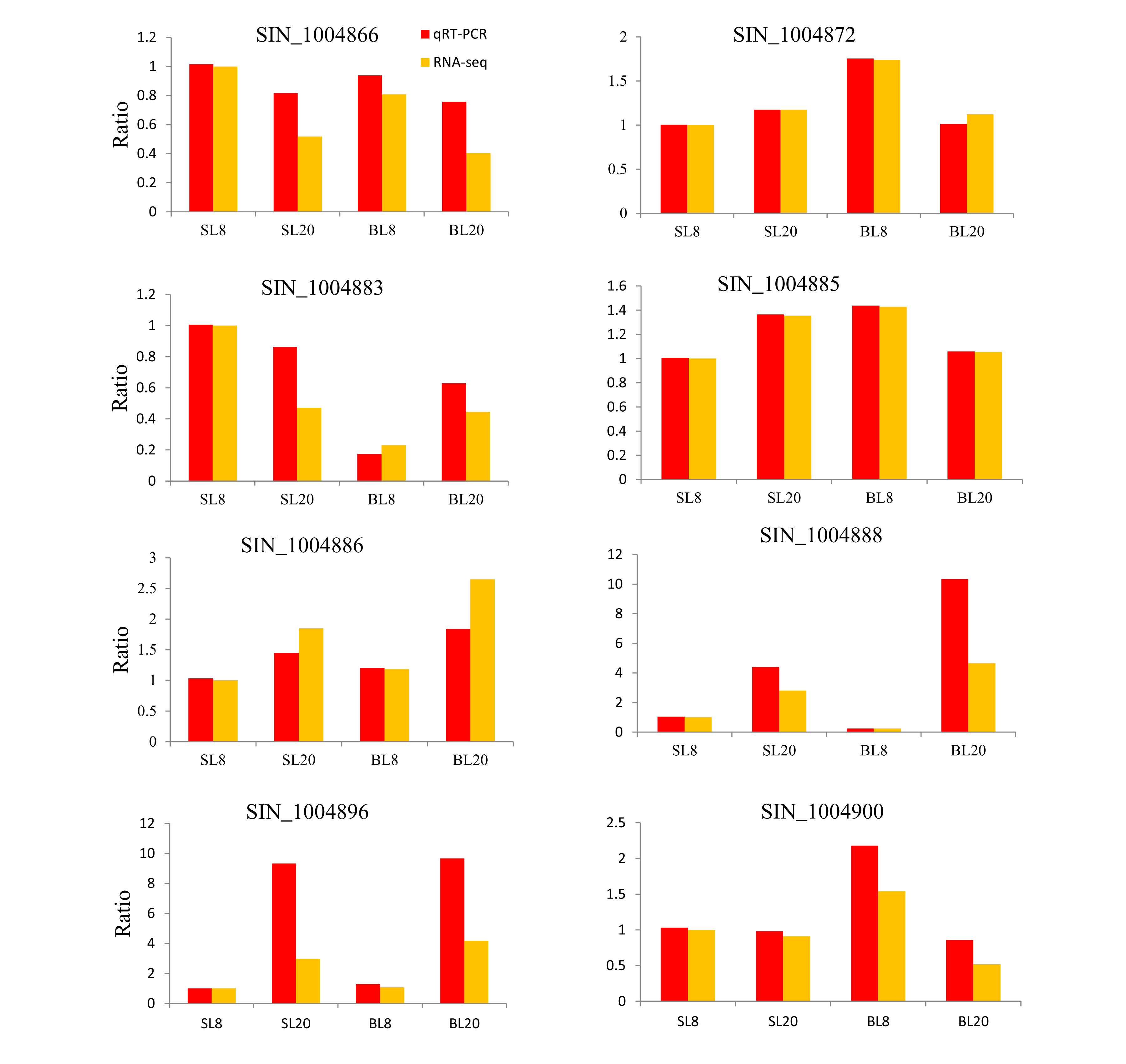

Supplement: Supplementary Figure 1 — Comparison of the relative expression abundance measured by qRT-PCR and RNA-seq for 8 selected genes between BL and SL at different stages. [file Image_1.TIF]
